# Genetic factors and symptom dimensions associated with antidepressant treatment outcomes: clues for new potential therapeutic targets?

**DOI:** 10.1007/s00406-024-01873-1

**Published:** 2024-08-27

**Authors:** Alfonso Martone, Chiara Possidente, Giuseppe Fanelli, Chiara Fabbri, Alessandro Serretti

**Affiliations:** 1 Department of Biomedical and Neuromotor Sciences, University of Bologna, Viale Carlo Pepoli 5, 40123 Bologna, Italy; 2 Department of Human Genetics, Radboud University Medical Center, Nijmegen, The Netherlands; 3 Donders Institute for Brain, Cognition and Behaviour, Radboud University, Nijmegen, The Netherlands; 4 Department of Medicine and Surgery, Kore University of Enna, Enna, Italy; 5 Oasi Research Institute-IRCCS, Troina, Italy

**Keywords:** Major depression, Drug-gene interaction analysis, Pharmacogenomics, Drug targets, Treatment-resistant depression (TRD), Antidepressants

## Abstract

Treatment response and resistance in major depressive disorder (MDD) show a significant genetic component, but previous studies had limited power also due to MDD heterogeneity. This literature review focuses on the genetic factors associated with treatment outcomes in MDD, exploring their overlap with those associated with clinically relevant symptom dimensions. We searched PubMed for: (1) genome-wide association studies (GWASs) or whole exome sequencing studies (WESs) that investigated efficacy outcomes in MDD; (2) studies examining the association between MDD treatment outcomes and specific depressive symptom dimensions; and (3) GWASs of the identified symptom dimensions. We identified 13 GWASs and one WES of treatment outcomes in MDD, reporting several significant loci, genes, and gene sets involved in gene expression, immune system regulation, synaptic transmission and plasticity, neurogenesis and differentiation. Nine symptom dimensions were associated with poor treatment outcomes and studied by previous GWASs (anxiety, neuroticism, anhedonia, cognitive functioning, melancholia, suicide attempt, psychosis, sleep, sociability). Four genes were associated with both treatment outcomes and these symptom dimensions: *CGREF1* (anxiety); *MCHR1* (neuroticism); *FTO* and *NRXN3* (sleep). Other overlapping signals were found when considering genes suggestively associated with treatment outcomes. Genetic studies of treatment outcomes showed convergence at the level of biological processes, despite no replication at gene or variant level. The genetic signals overlapping with symptom dimensions of interest may point to shared biological mechanisms and potential targets for new treatments tailored to the individual patient’s clinical profile.

**Supplementary Information:**

The online version contains supplementary material available at 10.1007/s00406-024-01873-1.

## Introduction

Major depression disorder (MDD) is a leading cause of disability worldwide and ranks as the fourth leading cause of morbidity, according to the World Health Organization [1]. Estimates of the lifetime prevalence of depression range from 7 to 20% [2] and the 12-month prevalence can be as high as 5% [1].

Major guidelines recommend pharmacotherapy as first-line treatment for moderate to severe MDD [3, 4]. Antidepressants are among the most commonly prescribed medications worldwide [5] and have proven efficacy in reducing depressive symptoms. Currently, clinicians have access to several treatment options belonging to different classes of antidepressants, but none of them has clear evidence of superiority over the others and the remission rates with antidepressant therapy are still concerningly low [6]. Indeed, only  ~ 30% of patients achieves remission after the first treatment, and even after four trials of treatment the percentage of remission does not reach 70% [7, 8]. After each treatment failure, the chance of response to the next antidepressant decreases and the risk of treatment-resistant depression (TRD), which is commonly defined as the lack of response to at least two treatments, increases [9, 10]. The trial-and-error process of finding an effective antidepressant can be prolonged and demoralizing, leading to delayed recovery and potentially contributing to the chronicity of the disease. Additionally, lack of treatment response exposes patients to a range of distressing and debilitating side effects [11, 12], which can undermine the therapeutic alliance.

Therefore, understanding the individual factors that influence treatment response in MDD is of primary importance to improve outcomes related not only to depressive symptoms, but also quality of life and overall functioning. Treatment response has a hereditary basis—for example, a concordance of antidepressant response- has been demonstrated in affected members of the same family [13] and a significant single nucleotide polymorphism (SNP)-based heritability (*h*^*2*^_*SNP*_) was found by genome-wide association studies (GWASs) [14]. Clinical and socio-demographic variables also contribute to treatment outcomes [15, 16]. Certain risk factors, such as suicidality and comorbid anxiety, may have a genetic basis overlapping with that of treatment response; conversely, socio-demographic variables and clinical factors such as duration and severity of the depressive episode, may exert effects independent from the genetics involved in treatment outcomes [17].

Although MDD is conceptualized as a single disorder, its diagnosis is formulated from a combination of symptoms that present with considerable variability, as over 1000 unique symptom combinations can be observed [18]. This phenotypic variability likely reflects the biological and environmental heterogeneity among patients, and may be partly linked to the heterogeneity observed in treatment response. Strong evidence shows that certain symptoms tend to co-occur, identifying subtypes of depression [19]. This is the case, for example, of atypical depression, which is characterized by the presence of mood reactivity and reversed neurovegetative symptoms (e.g. increased appetite/weight and hypersomnia). These symptoms likely have similar pathophysiological correlates and share common polygenic liabilities [20, 21]. On the other hand, distinct symptom domains are expressions of the heterogeneous genetic architecture of depression [22]. Consistently, specific symptom profiles tend to exhibit greater responsiveness to particular medications because of their pharmacodynamic profiles, in line with recommendations by the Canadian Network for Mood and Anxiety Treatments (CANMAT) guidelines [4]. Furthermore, certain depressive symptom dimensions such as anxiety, hypersomnia, anhedonia, suicidality, are associated with poorer treatment response, suggesting shared genetics between specific depressive symptom profiles and treatment outcomes [18].

Therefore, the aims of this narrative review are to (1) summarize the genetic factors associated with treatment efficacy outcomes in MDD; and (2) to explore the overlap between these genetic factors and those involved in specific symptom dimensions previously associated with treatment outcomes in MDD. This approach can provide information useful to identify specific biomarkers for treatment personalization, with implications for future research and clinical practice. To reach the second aim, we first reviewed the literature to identify the genetic signals associated with MDD treatment outcomes, focusing on GWASs and whole exome sequencing studies (WESs), given the instability of results from candidate gene studies [23, 24]. Then, we investigated whether these genetic associations overlap with those of MDD treatment outcomes. The rationale of this approach is to contribute to interpreting the existing literature in terms of genetic factors associated with treatment outcomes that may also be linked to specific symptoms, thereby helping to dissect the biological and clinical heterogeneity of poor treatment response and suggesting new treatment targets specific to certain symptom dimensions rather than MDD overall.

## Methods

We searched PubMed, GWAS catalog (https://www.ebi.ac.uk/gwas/), GWAS atlas (https://atlas.ctglab.nl/), and medRxiv (https://www.medrxiv.org/) for original research articles on the following topics: (1) GWASs or WESs that investigated efficacy outcomes in MDD (response, remission, symptom improvement, or TRD, as defined in each original study); (2) the association between MDD treatment outcomes and specific depressive symptoms/dimensions; (3) GWASs that investigated the genetics of the symptom dimensions identified in (2).

When more GWASs were available in (3) for the same trait, we focused on the largest study demonstrating a significant *h*^*2*^_*SNP*_. We considered association signals at locus, gene, and gene set level. As the main topic of this work was to review the genetics of treatment outcomes in MDD, for studies in (1) we decided to present both genetic associations surviving multiple testing correction and suggestive association signals (p-value  <  5 × 10^–8^ and 5 × 10^–8^  ≤  p-value < 5 × 10^–6^ at locus level, respectively). Although this was not a systematic review, we aimed to provide a comprehensive consideration of GWASs and WESs relevant to MDD treatment outcomes.

Finally, based on the overlap between genes identified (either significant or suggestive associations) from the SNP- and gene-level analyses of treatment outcomes and those significatively associated with the clinical dimensions in (3), we conducted a gene-drug interaction analysis via www.DGIdb.org [25].

## Results

### Genetic associations with treatment outcomes

A total of 13 GWASs and one WESs were included in this review, and their main characteristics are summarized in Table 1. Most studies had relatively small sample size (i.e., < 10 K), with only three exceptions, including one large study with 154,433 participants [26]; only 6 studies demonstrated a significant *h*^*2*^_*SNP*_ of the outcome(s) of interest, with values generally around 0.8–0.10.

**Table 1:** Characteristics of the genetic studies included for treatment outcomes (**A**) and associated clinical dimensions (**B**)

A						
References	Phenotype	Sample size	Type of study	h^2^_SNP_	h^2^_SNP_ phenotype	Number of significant SNPs/genes/gene sets
Huang et al. 2023 [82]	% symptom change, response	421	GWAS	–	–	28 gene sets
Kang et al. 2023 [26]	TRD	154,433	GWAMA	0.02–0.04	TRD vs non-TRD	2 SNPs
Pain et al. 2022 [14]	Remission, % change in symptom severity	5,151—5,218	GWAMA	0.13	Remission vs non-remission (mega-GREML)	2 genes
				0.396	Remission vs non-remission (meta-GREML)	
				−0.018 (NS)	Percentage improvement (mega-GREML)	
				0.215	Percentage improvement (meta-GREML)	
Fabbri et al. 2021 [83]	TRD	16,372	GWAS	0.08	TRD vs non-TRD	–
Kang et al. 2020 [84]	Remission	155–511	WES	–	–	11 genes, 53 gene sets
Li et al. 2020 [85]	Response, TRD	4,005–25,506	GWAS, GWAMA	0.08	TRD vs non-TRD	3 SNPs, 4 genes
				0.01 (NS)	SNRI response vs. non-response	
				0.04 (NS)	SSRI response vs non-response	
				0.07 (NS)	NDRI response vs. non-response	
Wigmore et al. 2020 [34]	TRD, stage of resistance	3,452–4,213	GWAS, GWAMA	0.27*	Stage of resistance	–
				0.60*	TRD vs non-TRD	
Fabbri et al. 2019 [86]	TRD, response, % symptom change	759–3,225	GWAS, GWAMA	–	–	2 gene sets
Fabbri et al. 2018 [87]	% symptom change, remission	1,422–2,145	GWAS, GWAMA	–	–	2 SNPs, 1 gene, 2 gene sets
Li et al. 2016 [88]	Response, TRD	4,536–9,688	GWAMA	0.11 (NS)	TRD vs non-TRD	1 SNPs, 18 gene sets
				0.12 (NS)	(Es)citalopram response vs non-response	
				0.05 (NS)	SSRI response vs non-response	
				−0.05 (NS)	NDRI response vs non-response	
Hunter et al. 2013 [89]	Sustained response	1,116	GWAS	–	–	7 genes, 8 gene sets
Uher et al. 2013 [89]	% symptom change, remission	1,354–2,256	GWAMA	–	–	2 SNPs
Tansey et al. 2012 [90]	% change in symptom severity	568–2,897	GWAS, GWAMA	–	–	–
Uher et al. 2010 [91]	% change in symptom severity	312–706	GWAS	–	–	1 SNP

B					
References	Phenotype	Sample size	Type of study	h^2^_SNP_	Number of significant SNPs/genes/gene sets
Friligkou et al. 2024 [92]	Anxiety	1,096,458	GWAMA, TWAMA, PWAMA	0.05	42 SNPs, 118 genes
Nagel et al. 2018 [93]	Neuroticism	449,484	GWAMA	0.05–0.10	145 SNPs, 599 genes, 7 gene sets
Ward et al. 2019 [94]	Anhedonia	375,275	GWAS	0.06	11 SNPs
Hatoum et al. 2023 [95]	Executive functions	427,037	GWAS	0.09–0.10	342 SNPs, 353 genes, 12 gene sets
Cai et al. 2015 [96]	Melancholic features	4,509	GWAS	–	29 SNPs
Docherty et al. 2020 [97]	Suicide attempt	518,612–958,896	GWAMA	0.06	38 SNPs, 37 genes, 519 gene sets
Barkhuizen et al. 2020 [98]	Psychotic experiences	116,787–117,794	GWAS	0.07–0.10	104 SNPs
Bralten et al. 2021 [99]	Sociability	342,461	GWAS	0.06	19 SNPs, 56 genes, 8 gene sets
Goodman et al. 2024 [100]	Sleep health score	413,904	GWAS	0.07–0.15	401 SNPs, 588 genes, 860 gene sets

*AD* antidepressant, *GWAMA* genome-wide association meta-analysis, *GWAS* genome-wide association study, *h*^*2*^_*SNP*_ SNP-based heritability, *PWAS* proteome-wide association study, *SNP* single-nucleotide polymorphism, *TRD* treatment-resistant depression, *TWAS* transcriptome-wide association study, *WES* whole exome sequencing, *NS* non-significant

*Pedigree-based heritability estimates and not h^2^_SNP_

At locus level, 11 genome-wide significant associations were identified, spanning across multiple genes, including *ITGA9*, *NRXN3*, *UST*, *MECOM*, *FTO*, and *MCHR1* (Table 2A). Additional suggestive signals were identified, including for example *LINGO2*, *CACNA1C*, *PRG3*, *ITGA1*, *EPHB1* and *SLC27A1* genes (Supplementary Table 1). However, we underline that these non-significant results have unclear relevance and were not replicated across studies.

**Table 2:** Genome-wide significant variants (**A**), genes (**B**), and gene sets (**C**) associated with AD treatment outcome

A			
Phenotype/outcome	Locus/variant significant	Gene(s) corresponding	Sources
AD response	rs12054895	Intergenic	Uher et al. 2013 [101]
	rs17651119	*MYO10*	
AD response	rs1908557	Intergenic, between *GPRIN3* and *SNCA* (*SNCA*)	Li et al. 2016 [88]
% symptom change	rs116692768	*ITGA9*	Fabbri et al. 2018 [87]
	rs76191705	*NRXN3*	
% symptom change	rs2500535	*UST*	Uher et al. 2010 [91]
AD response	rs4955665	*MECOM*	Li et al. 2020 [85]
	rs4884091	*RNF219*-*AS1*	Li et al. 2020 [85]
TRD	rs150245813	Intergenic, several eQTLs (*LTB*)	Li et al. 2020 [85]
	rs56313538	*FTO*	Kang et al. 2023 [26]
	rs133082	*MCHR1*	

B		
Phenotype/outcome	Genes	Source
Remission	*BRPF3, CGREF1, COMT, LZTS3, MAP1A, MEPCE, PFAS, PPFIBP1, PRNP, SLC25A40, ST3GAL5*	Kang et al. 2020 [84]
	*DHX8, ETV4*	Pain et al. 2021 [14]
AD response	*B9D1, C11ORF85, C9ORF16, EPN2, GPHA2, LCN2, PPP2R5B*	Hunter et al. 2021 [89]
% symptom change	*OR4K2*	Fabbri et al. 2018 [87]
AD response	*ADAMTS5*	Li et al. 2020 [85]
TRD	*LST1, LTB, NCR3*	Li et al. 2020 [85]

C				
Outcome	Function	Gene set		Source
AD response	Differentiation	GGCCAGT_MIR193A_MIR193B		Li et al. 2016 [88]
		HALLMARK_ADIPOGENESIS		Li et al. 2016 [88]
Sustained response	Endocrine	BIOCARTA_MPR_PATHWAY		Hunter et al. 2013 [89]
		hsa04940	Type I diabetes mellitus	Hunter et al. 2013 [89]
AD response	Immunity	GO:0002460	Adaptive immune response based on somatic recombination of immune receptors built from immunoglobulin superfamily domains	Huang et al. 2023 [82]
		WP2328	Allograft Rejection	Huang et al. 2023 [82]
		GO:0019724	B cell mediated immunity	Huang et al. 2023 [82]
		GO:0006956	Complement activation	Huang et al. 2023 [82]
		WP545	Complement Activation	Huang et al. 2023 [82]
		GO:0006957	Complement activation, alternative pathway	Huang et al. 2023 [82]
		GO:0006958	Complement activation, classical pathway	Huang et al. 2023 [82]
		R-HSA-166658	Complement cascade	Huang et al. 2023 [82]
		GO:0002455	Humoral immune response mediated by circulating immunoglobulin	Huang et al. 2023 [82]
		GO:0016064	Immunoglobulin mediated immune response	Huang et al. 2023 [82]
		GO:0002449	Lymphocyte mediated immunity	Huang et al. 2023 [82]
		GO:0005579	Membrane attack complex	Huang et al. 2023 [82]
		GO:0030449	Regulation of complement activation	Huang et al. 2023 [82]
		R-HSA-977606	Regulation of Complement cascade	Huang et al. 2023 [82]
		GO:0002920	Regulation of humoral immune response	Huang et al. 2023 [82]
		R-HSA-166665	Terminal pathway of complement	Huang et al. 2023 [82]
		CMP_7107	TNF pathway	Hunter et al. 2013 [89]
		hsa04612	KEGG_ANTIGEN_PROCESSING_AND_PRESENTATION	Hunter et al. 2013 [89]
AD response		PID_CDC42_REG_PATHWAY	PID_CDC42_REG_PATHWAY	Li et al. 2016 [88]
AD response	Metabolism	ko00460	Cyanoamino_acid_metabolism	Li et al. 2016 [88]
		hsa00534	KEGG_GLYCOSAMINOGLYCAN_BIOSYNTHESIS_HEPARAN_SULFATE	Li et al. 2016[88]
		hsa00430	KEGG_TAURINE_AND_HYPOTAURINE_METABOLISM	Li et al. 2016 [88]
		hsa00565	LIPID_HOMEOSTASIS	Li et al. 2016 [88]
Sustained response	Neurodegeneration	hsa05010	KEGG_ALZHEIMERS_DISEASE	Hunter et al. 2013 [89]
AD response	Neurotransmission	GO:0019228	Neuronal action potential	Huang et al. 2023 [82]
		GO:0019226	Transmission of nerve impulse	Huang et al. 2023 [82]
Sustained response		hsa04720	KEGG_LONG_TERM_POTENTIATION	Hunter et al. 2013 [89]
AD response		hsa04730	KEGG_LONG_TERM_DEPRESSION	Li et al. 2016 [88]
AD response	Nuclear	GO: 0044427	Chromosomal part	Fabbri et al. 2018 [87]
		GO:0005694	Chromosome pathway	Fabbri et al. 2018 [87]
AD response		HALLMARK_E2F_TARGETS		Li et al. 2016 [88]
	Positional geneset	chr16p12		Li et al. 2016 [88]
		chr22q11		Li et al. 2016 [88]
		chr8p22		Li et al. 2016 [88]
AD response	Signaling	R-HSA-1296052	Ca2 + activated K + channel	Huang et al. 2023 [82]
		GO:0015269	Calcium-activated potassium channel activity	Huang et al. 2023 [82]
		R-HSA-373076	Class A/1 (Rhodopsin-like receptors)	Huang et al. 2023 [82]
		GO:0005513	GOBP_DETECTION_OF_CALCIUM_ION	Huang et al. 2023 [82]
		(R-HSA-418594)	G alpha (i) signaling events	Huang et al. 2023 [82]
		R-HSA-500792	GPCR ligand binding	Huang et al. 2023 [82]
		WP455	GPCRs, Class A Rhodopsin-like	Huang et al. 2023 [82]
Sustained response		CMP_6680	G alpha q pathway	Hunter et al. 2013 [89]
		PAHS-043A	WNT_SIGNALING	Hunter et al. 2013 [89]
AD response		R-HSA-418038	REACTOME_NUCLEOTIDE_LIKE_PURINERGIC_RECEPTORS	Li et al. 2016 [88]
		GO:0005070	SH3_SH2_ADAPTOR_ACTIVITY	Li et al. 2016 [88]
	Stress response	GO:0019835	Cytolysis	Huang et al. 2023 [82]
		BIOCARTA_VEGF_PATHWAY		Li et al. 2016 [88]
AD response	Structural	GO:0098797	Plasma membrane protein complex	Huang et al. 2023 [82]
		GO:0046930	Pore complex	Huang et al. 2023 [82]
AD response		GO:0060090	MOLECULAR_ADAPTOR_ACTIVITY	Li et al. 2016 [88]
		GO:0030674	PROTEIN_BINDING_BRIDGING	Li et al. 2016 [88]
TRD	Metabolism	hsa00071	KEGG_FATTY_ACID_METABOLISM	Li et al. 2016 [88]
	Signaling	GO:0043949	GOBP_REGULATION_OF_CAMP_MEDIATED_SIGNALING	Fabbri et al. 2019 [86]
TRD	Nuclear	GO:0000183	GOBP_NUCLEOLAR_CHROMATIN_ORGANIZATION	Fabbri et al. 2019 [86]

*AD* antidepressant, *TRD* treatment resistant depression

A total of 25 genes were associated with the outcomes in the gene-level analyses, including *LZTS3*, *PRNP*, *OR4K2*, *PPFIBP1*, and *GPHA2* (see Table 2B for all results). Suggestive associations were identified, including *ADGRG5*, *MAP3K2*, the solute carrier genes *SLC17A4* and *SLCO3A1*, the glutamate receptor gene *GRM3*,, and genes encoding for many zinc finger proteins (Supplementary Table 2). However, none of these were replicated across studies.

Noteworthy, both suggestive and significant association findings had some consistency in terms of biological processes involved at the pathway level, showing an involvement of the immune system (e.g.: *NCR3*, *LST1*, and *LCN2*), synaptic transmission and dendritic spine formation (e.g.: *PPFIBP1*, *PRNP*, *LZTS3*, and *NRXN3*), neurogenesis and differentiation (e.g.: *NRXN3*, *MECOM*, *CGREF1* and *MAP1A*), and regulation of transcription and gene expression (e.g.: *DHX8*, *MECOM*, *ETV4*, *MEPCE*, and *PFAS*). These observations are supported by the findings from gene set enrichment analyses (GSEAs). In particular, while no enrichment for specific gene sets was replicated across studies, GSEAs showed that many gene-sets are involved in or regulate similar biological processes. For example, signal transduction (Rhodopsine-like receptors A/1 R-HSA-373076 and Calcium-activated potassium channel activity GO:0015269); gene expression and nuclear functions (Chromosomal part GO:0044427 and Chromosome pathway GO:0005694), neurotransmission and synapse activity (Neuronal action potential GO:0019228, Transmission of nerve impulse GO:0019226, Long term potentiation hsa04720), immune function (Lymphocyte mediated immunity GO:0002449) ( Table 2C and Supplementary Table 3).

### Genetic associations with symptom dimensions

Nine symptom dimensions were identified as associated with poor treatment outcomes in MDD and studied by previous GWASs showing significant *h*^*2*^_*SNP*_, namely: anxiety symptoms, neuroticism (including symptoms of apathy, worthlessness, guilt, loneliness, and excessive worry), anhedonia, cognitive functioning, melancholia, suicide attempt, psychotic symptoms, sleep symptoms, and sociability. No GWASs showed significant *h*^*2*^_*SNP*_ for other symptoms also associated with poor therapeutic outcomes, such as irritable mood, inner tension, dissociative symptoms, and reverse or typical neurovegetative symptoms (with the exception of sleep-related symptoms) [15, 16, 27, 28].

The main characteristics of the included studies are summarised in Table 1, whilst full details and results are reported in the Supplementary Tables 1, 2, and 3. The selected GWASs were performed on large samples, in the range of 500 K participants, and all reported multiple significant genetic associations with the traits of interest (Table 1 and Supplementary Tables 1 and 2).

Given the focus of this review, a comprehensive description of these results is beyond our aims, while we were interested in describing the potential overlap with the genetic associations found for MDD treatment outcomes. Among the genes significatively associated with treatment outcomes, four overlapped with those associated with symptom dimensions, more precisely with anxiety (*CGREF1*), neuroticism (*MCHR1*) and sleep (*FTO*, *NRXN3*) (Table 3, in bold).

**Table 3:** Genes associated with AD treatment outcomes overlapping with genetic signals associated with symptom dimensions

Gene	Outcome	Symptom dimension	Drug
**FTO**	TRD	Sleep (SHS-PC1, PC3, PC4)	INTERFERON ALFA-2A
			RIBAVIRIN
			INTERFERON ALFA-2B
			ATENOLOL
			AZATHIOPRINE
			MERCAPTOPURINE
			BISANTRENE
			HYDROCHLOROTHIAZIDE
**MCHR1**	TRD	Neuroticism	BMS-830216
			SB-568849
			SNAP-7941
			T-226296
**NRXN3**	% symptom change	Sleep (SHS-ADD/PC1/PC2)	None
**CGREF1**	Remission	Anxiety	None
GRM3	AD response	Neuroticism, executive functions	LY404039
			OLEOYL-ESTRONE
			MGS-0210
			DECOGLURANT
			HEROIN
			LY2140023
			POMAGLUMETAD METHIONIL
			LY2969822
			RISPERIDONE
			DECOGLURANT
CACNA1C	TRD	Executive functions	NICARDIPINE HYDROCHLORIDE
			NIFEDIPINE
			CILNIDIPINE
			NICARDIPINE
			ELPETRIGINE
			CALCIUM CHANNEL BLOCKER
			DILTIAZEM MALATE
			DILTIAZEM HYDROCHLORIDE
			ISRADIPINE
			MANIDIPINE
			DENATURED ETHANOL
			NILVADIPINE
			SULOCTIDIL
			ARVERAPAMIL
			CELECOXIB
			CLEVIDIPINE
			NIMODIPINE
			VERAPAMIL
			BENIDIPINE
			NISOLDIPINE
			AZD1305
			PREGABALIN
			GABAPENTIN
			LACIDIPINE
			RITODRINE
			LERCANIDIPINE HYDROCHLORIDE
			NITRENDIPINE
			AMLODIPINE BENZOATE
			CITALOPRAM
			MEPIRODIPINE
			ATENOLOL
			IMAGABALIN
			LEVAMLODIPINE MALEATE
			HALOPERIDOL DECANOATE
			IBUTILIDE
			AMLODIPINE MALEATE
			RAUWOLFIA SERPENTINA (USP)
			VALPROIC ACID
			DRONEDARONE HYDROCHLORIDE
			GABAPENTIN ENACARBIL
			CINNARIZINE
			ATAGABALIN
			TERODILINE HYDROCHLORIDE
			BEPRIDIL HYDROCHLORIDE
			AZELNIDIPINE
			AMLODIPINE BESYLATE
			PHLOROGLUCINOL
			FELODIPINE
CTNNA3	AD response	Anhedonia	ANTIDEPRESSANT AGENT
EPHB1	TRD	Neuroticism, anhedonia, executive functions	RECOMBINANT FIBROBLAST GROWTH FACTOR 2
			VANDETANIB
			HESPERADIN
			PROGESTERONE
ARFGEF2	AD response	Executive functions	None
USMG5 (ATP5MK)	AD response	Neuroticism, sleep	None
PKHD1	TRD	Neuroticism	None
PRSS35	AD response	Sleep (SHS-PC2)	None
SCG3	TRD	Executive functions	None
AVL9	TRD	Sleep (SHS-PC3)	None
SATB1-AS1	TRD	Anxiety	None
CAMKMT	TRD	Sleep (SHS-PC3)	None
GNAZ	AD response	Sleep (SHS-PC2)	None
MTRNR2L9	SNRI response	Sleep (SHS-PC2)	None
LINC01360	AD response	Anxiety	None
RTDR1	AD response	Sleep (SHS-PC2)	None
SGCZ	TRD	Neuroticism, executive functions, sociability, sleep	None
LINGO2	Remission	Neuroticism	None

For selecting genes associated with treatment outcomes, we considered associations at variant level annotated with the corresponding gene or at gene level, either significant or suggestive (those which were significant are in bold), as explained in the methods paragraph

*AD* Antidepressant, *SHS-ADD* sleep health score-additive, *SHS-PC* sleep health score-principal component, *TRD* treatment resistant depression

When considering also genes suggestively associated with treatment outcomes, additional signals were found in common with anxiety, anhedonia, executive functions, and sociability, as well as more overlapping genes for neuroticism and sleep (Table 3).

At gene set level, exact matches were found only with sleep, involving gene sets related to synaptic activity (hsa04730, hsa04730), G alpha signalling (R-HSA-418594), taurine/hypotaurine metabolism (hsa00430), immune response (BIOCARTA_VEGF_PATHWAY, hsa04612), and Alzheimer’s disease (hsa05010). However, even if no exact gene set overlaps were found between MDD treatment outcomes and other symptom dimensions, we identified patterns of possible overlap in some biological mechanisms involved. For example, gene sets associated with neuroticism, executive functions, and suicide attempts are predominantly related to neurogenesis and neurotransmission. Similarly, also other biological processes are involved both in MDD treatment outcome and some of the clinical dimensions, like synaptic plasticity (executive functions), immune response (suicide attempt, sleep), and nucleic acid and gene expression (suicide attempt) (Supplementary Table 3).

### New therapeutic targets for treating symptom dimensions associated with poor response?

Based on the overlap discussed in the previous paragraph, we conducted a gene-drug interaction analysis. Of the four significant overlapping genes, no compounds were found to interact with either *NRXN3* or *CGREF1*, while interactions were found only for the melanin concentrating hormone receptor 1 (*MCHR1*) and the fat mass and obesity associated gene (*FTO*, also known as *ALKBH9*). MCHR1, also known as SLC1, is a G-protein coupled receptor (GPCR) which binds the melanin-concentrating hormone (MCH, or PMCH) and inhibits cAMP accumulation while stimulating intracellular calcium influx. MCH is likely involved in the regulation of feeding behaviour, mood, sleep–wake cycle and energy balance [29]. Four still non-approved compounds resulted to interact with *MCHR1*, of which SNAP-7941 is the lead compound of MCHR1-inhibitors and displayed promising anxiolytic, antidepressant, and anorectic effects, even though not replicated in clinical trials [30]. Another MCHR1 antagonist, BMS-830216, is currently in phase 2 for the treatment of obesity [31].

Concerning *FTO*, eight compounds were found, all already approved as antineoplastics (INFɑ-2A, INFɑ-2B, mercaptopurine, and bisantrene), antiviral (ribavirin), antiarrhythmic (atenolol), antihypertensive (atenolol, hydrochlorothiazide), and disease-modifying antirheumatic drugs (azathioprine). *FTO*’s exact physiological function is yet to be uncovered; however, it is a non-heme iron enzyme located in the nucleus and likely related to growth, development, BMI, obesity, and type 2 diabetes mellitus [32].

When considering the overlap with genes associated with poor treatment outcome at a suggestive level, interesting gene-drug interactions were found for *GRM3* with risperidone and two selective mGluR2/3 agonists (LY2969822 and LY404039, and the corresponding prodrug of the latter LY2140023); and *CACNA1C* with haloperidol, citalopram, valproate, and gabapentinoids. For all gene-drug interactions, see Table 3.

## Discussion

The identification of genetic factors modulating MDD treatment outcomes has been challenging and led to few clinical applications, limited to genes involved in drug metabolism [33]. In the 50 years after the first evidence of a substantial heritability coming from family genetic studies [33], many studies focused on candidate genes, with minor and mainly unreplicated findings. In the last 10 years, GWASs produced more interesting findings, due to a more extensive coverage of the genome and larger samples, as discussed in this review. To date, it has been estimated that genetics may account for up to 60% of the variance in treatment resistance according to pedigree-based heritability [34].

However, the polygenic nature of MDD treatment outcomes and the relatively limited size of most samples resulted in scattered results which do not generally overlap, at least at SNP or gene level. The redundancies among the pathways modulating antidepressant outcomes and the heterogeneity of depressive symptoms are likely involved in the discrepancy of results at SNP and gene level [35–38]. The approach used in the present review aimed to partially overcome these issues, by integrating the genetic signals associated with antidepressant outcomes and specific symptom dimensions of clinical relevance, and by extending the analysis to pathways, pointing out potential mechanisms involved in treatment resistance and possible treatment targets.

Both at variant/gene level and gene set level, treatment outcomes were linked to gene expression regulation, central nervous system (CNS) development, synaptic plasticity, and immune system activity. One speculative interpretation of these results is that anomalies in the regulation of gene expression concur with abnormal CNS development (from tissue differentiation to synapse formation) and with an aberrant brain-immunity interplay, resulting in increased risk of developing more severe and less treatment-responsive MDD.

We identified four genes that were significantly associated with both treatment outcomes and the clinical dimensions of interest, namely *CGREF1* (anxiety), *MCHR1* (neuroticism), *FTO* and *NRXN3* (sleep). *NRXN3* (neurexin 3) encodes for a surface protein acting as cell adhesion molecule-receptor and it is likely involved in synaptic plasticity [39]. Other than with treatment response, it was also associated with sleep health (Supplementary Tables 1 and 2), suggesting a protective function on brain physiological activity. Polymorphisms in this gene have been linked to obesity and substance use disorders [40, 41], suggesting an influence on impulse control. This increases the interest towards this gene and the possibility to target/modulate it by future therapies, despite currently there are no known compounds/molecules targeting *NRXN3*.

*FTO* (fat mass and obesity-associated protein) and *MCHR1* (melanin concentrating hormone receptor 1) were both significantly associated with TRD and with sleep and neuroticism, respectively. Both are involved in energy homeostasis, but *FTO* acts more on a cellular level regulating adipogenesis and fat mass [32, 42–44], while *MCHR1* is involved in the regulation of feeding behaviour and energy balance and has known effects on mood and sleep–wake cycle [45]. Variants in *MCHR1* have been linked to a reduced expression in the dorsolateral prefrontal cortex [46] and are in linkage disequilibrium with another variant recently linked to an increased risk of bipolar disorder [47]. Several drugs interact with *FTO* but, as far as we know, no specific compound has been developed or studied specifically. Interestingly, *FTO* also maps to a genomic region showing significant local genetic correlation between MDD and type 2 diabetes mellitus and obesity, suggesting that *FTO* could be implicated in the shared etiopathogenesis between MDD and insulin resistance-related conditions [48]. We found *FTO* to interact with antihypertensive drugs, i.e. hydrochlorothiazide (a diuretic) and atenolol (a β-blocker). Angiotensin agents, calcium-channel blockers, and β-blockers (but not diuretics) have been recently associated with decreased rates of depression [49]. On the other hand, *MCHR1* has been investigated and promising results were found for some compounds [50, 51], but results were not replicated [30].

*CGREF1* (cell growth regulator with EF-hand domain 1) inhibits cell proliferation, despite its function has not been fully elucidated [52]. We found that this gene was associated with remission to antidepressants and anxiety (Supplementary Table 2), but it was implicated in other traits as well by previous GWASs, such as brain measures, risk-taking behaviours, wellbeing, and educational attainment [53–57]. Therefore, *CGREF1* may be involved in the modulation of multiple but likely connected phenotypes, which are relevant for antidepressant effects. Currently, there are no known compounds that target *CGREF1*.

Other genes were not significantly associated with antidepressant outcomes, however suggestive findings were reported (Supplementary Tables 1–3). Among these genes, we outline *GRM3*, [58], *SLCO3A1*, *LINGO1* and *LINGO2*. *EPHB1* (Supplementary Table 1) regulates chemotaxis and proliferation of neural progenitors in the hippocampus [58], a well known region for mood disorders physiopathology and AD efficacy [59, 60]. It was first identified as potentially involved in antidepressant response by one of the first GWASs in this field, which reported a suggestive association with a SNP located downstream of this gene [61]; however, this GWAS was not formally included in the present review, as it included both unipolar (MDD) and bipolar depression, while this work was focused on MDD. This gene was also associated with several symptom dimensions of interest, including neuroticism, anhedonia, and executive functions. *EPHB1* has a key role in axon guidance and, with other ephrin-B receptors, is involved in the development and maturation of dendritic spine and synapse formation [58]. To date, no specific drug exists to selectively target *EPHB1*, however it interacts with progesterone [25], which has been proved to modulate the expression of γ-aminobutyric acid type-A receptors (GABA_A_R) via its metabolite allopregnanolone [62]. Noteworthy, brexanolone, a synthetic allopregnanolone analogous, is approved for the treatment of postpartum depression [63].

*GRM3* represents an interesting finding. Unlike NMDA (N-Methyl-D-aspartic acid) and AMPA (α-amino-3-hydroxy-5-methyl-4-isoxazolepropionic acid) receptors, glutamate metabotropic receptors are G-protein coupled receptors (GPCR) with more complex effects, involved in synapse plasticity, for example long-term depression (LTD) at excitatory synapses [64]. *GRM3* was associated with neuroticism and executive functions (Supplementary Table 2). Thus, compounds acting at this site may hypothetically be beneficial for patients experiencing a depressive episode with a sense of guilt and worthlessness, interpersonal sensibility, and/or cognitive symptoms. To date, the only drug that seems to interact with *GRM3* is risperidone, a second-generation antipsychotic (SGA) used as adjunctive therapy in psychotic depression or as augmentation to antidepressants in TRD [65]. Other compounds targeting glutamate receptors are under study for the treatment of schizophrenia [66] and depressive disorders, such as MGS-0210 [67], which is a selective metabotropic glutamate receptor antagonist with antidepressant-like activity [68].

Even if only suggestive, the association of the organic anion transporter *SLCO3A1* with remission after treatment may suggest a role of endogenous organic anions, vasopressin and prostaglandins in MDD outcome [69, 70]. Noteworthy, *SLCO3A1* is significatively expressed in the CNS, especially in oligodendrocytes [70, 71] but also in neurons and grey matter glial cells [69, 72]. However, *SLCO3A1* activity is not yet fully understood and new interactions have been found with other exogenous compounds [73], such as modulation of its expression levels by valproic acid [74].

The role of energy metabolism in mood disorders is well known, as discussed above for *FTO* and *MCHR1,* and there is an increasing consensus on the involvement, for example, of glucidic metabolism in brain disorders [75, 76]. We observed significant association with many genes involved in feeding behaviour and sleep–wake cycle. *LINGO1* and *LINGO2* were associated with symptom remission and neuroticism, and are likely involved in synapse assembly [77], other than being associated with BMI [78]. No drug exists to date targeting these genes, but they are modulated by resveratrol and vitamin D [79] and they may represent putative targets for future complementary treatments, for example in patients with higher levels of neuroticism and BMI [80, 81].

This review provides a comprehensive overview of GWASs and WESs on treatment outcomes in MDD, also leveraging an innovative, clinically-oriented approach to explore the complex genetics of MDD treatment outcomes. By integrating genetic signals associated with MDD treatment outcomes and specific depressive symptom dimensions, our approach may pave the way for developing targeted treatments for non-responsive patients exhibiting specific symptom profiles. However, several limitations should be acknowledged. Firstly, we did not perform statistical analyses to test the genetic overlap between treatment outcomes and the symptom dimensions of interest; however, this was beyond the aims of this paper, being this work intended as a review of the literature. A common limitation of the included GWASs on MDD treatment outcomes was the relatively small sample size, and the resulting limited power to detect genome-wide significant associations and to replicate findings across studies. Last but not least, pharmacogenetic findings and targets identified by gene-drug interactions need functional validation to assess their potential clinical relevance and applicability. This consideration outlines the importance of using complementary and integrated research approaches, for example in vitro/in vivo models evaluating compound properties and activity.

In conclusion, this review presents significant insights into the genomics of treatment outcomes in MDD, highlighting the existence of genetic factors overlapping with specific clinical dimensions that are in turn associated with poor treatment outcomes. We prioritised four genes, including *CGREF1*, *MCHR1*, *FTO*, and *NRXN3*, which are linked to both MDD treatment outcomes and relevant clinical dimensions. These findings highlight the potential for developing new treatments that target specific depressive symptom dimensions, contributing to the advancement of precision psychiatry.

## Supplementary Information

Below is the link to the electronic supplementary material.

Supplementary file1 (XLSX 459 KB)
